# Evaluation of Different Procedures for Titanium Dental Implant Surface Decontamination—In Vitro Study

**DOI:** 10.3390/bioengineering11040326

**Published:** 2024-03-28

**Authors:** Ante Jordan, Igor Smojver, Ana Budimir, Dragana Gabrić, Marko Vuletić

**Affiliations:** 1 Department of Oral Surgery, Dental Polyclinic Zagreb, 10000 Zagreb, Croatia; 2 St. Catherine Specialty Hospital, 10000 Zagreb, Croatia; ismojver@gmail.com; 3 Department of Clinical Microbiology, Infection Prevention and Control, University Hospital Centre Zagreb, University of Zagreb, 10000 Zagreb, Croatia; abudimir@kbc-zagreb.hr; 4 Department of Oral Surgery, School of Dental Medicine, University Hospital Centre Zagreb, University of Zagreb, 10000 Zagreb, Croatia; dgabric@sfzg.hr (D.G.); mvuletic@sfzg.hr (M.V.)

**Keywords:** dental implant, biofilm, *Acinetobacter baumannii*, *Staphylococcus aureus*, periimplantitis, decontamination, chlorhexidine, chitosan, electrolytic cleaning

## Abstract

Polymicrobial biofilm removal and decontamination of the implant surface is the most important goal in the treatment of periimplantitis. The aim of this study is to evaluate the efficacy of four different decontamination methods for removing *Acinetobacter baumannii* and *Staphylococcus aureus* biofilms in vitro. Seventy-five dental implants were contaminated with a bacterial suspension and randomly divided into five groups (*n* = 15): the negative control group, which received no treatment; the positive control group, treated with 0.2% chlorhexidine; group 1, treated with a chitosan brush (Labrida BioCleanTM, Labrida AS, Oslo, Norway); group 2, treated with a chitosan brush and 0.2% chlorhexidine; and group 3, treated with a device based on the electrolytic cleaning method (GalvoSurge, GalvoSurge Dental AG, Widnau, Switzerland). The colony-forming unit (CFU) count was used to assess the number of viable bacteria in each sample, and statistical analyses were performed. When compared to the negative control group, all the decontamination methods reduced the CFU count. The electrolytic cleaning method decontaminated the implant surface more effectively than the other three procedures, while the chitosan brush was the least effective. Further research in more realistic settings is required to assess the efficacy of the decontamination procedures described in this study.

## 1. Introduction

Modern dental implantology is a reliable and predictable method of treating partial or complete tooth loss [1,2]. Despite the high success rate, the occurrence of complications, especially of periimplantitis, can cause the loss of the implant and consequent negative functional, emotional and financial impacts on the patient [3,4,5].

Periimplantitis is an inflammation of the soft and hard tissue surrounding an osseointegrated dental implant with a prevalence of about 20% in patients who have undergone implant therapy [1,6,7].

Biofilm on the exposed surface of the implant is the main etiological factor of periimplantitis [6,8,9]. Biofilm formation is a complicated multistep process involving the colonization of bacteria inhabited around dental implants, teeth and other parts of the oral cavity [9,10]. The oral biofilm associated with the occurrence of periimplantitis is characterized by a large microbial diversity, and no specific or unique bacteria have been identified that would be exclusively present in the biofilm of implants with periimplantitis [6,8]. The existence of a biofilm on the surface of the implant for a long period of time triggers the host’s immune response and the onset of inflammation [7,9]. This was confirmed in the research made by Choe et al., in which they state that the bacteria *Acinetobacter baumannii* and *Staphylococcus aureus* induced the production of inflammatory cytokines, inhibited osteogenesis and stimulated bone resorption around a titanium implant placed in the femur of a mouse [11].

One of the most demanding but also the most important tasks in the treatment of periimplantitis is the removal of biofilm and the prevention of new biofilm formation [3,5,7]. The presence of deep pockets in periimplantitis, difficult access to all implant surfaces, rough surfaces and implant threads can make the process of biofilm removal and decontamination challenging. This has led to research into different therapeutic protocols, biofilm removal methods and procedures for cleaning the contaminated implant surface [3,5,7].

Mechanical means such as curettes, sonic and ultrasonic instruments, air polishing devices and rotating titanium and chitosan brushes are used to remove hard and soft deposits from the implant surface [3,5]. It is recommended that the mentioned devices used during the cleaning change or damage the surface of the implant as little as possible. During the last few years, in order to deliver as little damage as possible to the implant surface, several types of lasers have been developed and several protocols of antimicrobial photodynamic therapy have been established [3]. The electrolytic cleaning method showed promising results in the elimination of bacteria from the rough surface of titanium implants [12,13,14,15,16]. It is a newer method of decontamination, and it is based on the application of a galvanic current to the implant [12,13]. A sodium formiate solution acting as an electrolyte is pumped by a device through a platinized ring acting as an anode and sprayed on the exposed and infected implant surface [12]. Electrolysis produces hydrogen cations that penetrate the biofilm. Hydrogen bubbles emerge on the implant surface and detach the biofilm from the surface [12,17]. Zipprich et al. showed that an electrolysis-based device removed bacteria from an implant surface more effectively in comparison to a diode laser, an air polishing device or a plasma therapy device [13].

In addition to mechanical means, as an additional measure in the treatment of periimplantitis, it is recommended to carry out surface decontamination by chemical means [18]. The most commonly used chemical agents for these purposes are sterile saline, hydrogen peroxide, citric acid, ethylenediaminetetraacetic acid, phosphoric acid and chlorhexidine gluconate [3]. 

Each of the mentioned methods has its advantages and disadvantages, and it has not been established that any of the examined procedures is superior to others [3,5,13,18,19]. 

The aim of this study was to investigate and compare the effectiveness of *A. baumannii* and *S. aureus* biofilm removal from the surface of titanium dental implants in vitro using a 0.2% chlorhexidine solution, a chitosan brush and an electrolytic cleaning device. To the best of our knowledge, there are no in vitro studies that have been carried out under the same conditions that have compared the effectiveness of the decontamination procedures assessed in this study.

## 2. Materials and Methods

This research was performed in vitro on 75 titanium dental implants (GC Aadva Standard Implants; GCTech.Europe GmbH, Breckerfeld, Germany) with a diameter of 4.0 mm and a length of 10 mm. All microbiological procedures were performed in the laboratory of the Department of Clinical Microbiology, Infection prevention and Control, University Hospital Centre Zagreb. The study was approved by the Ethics Committee of the School of Dental Medicine, University of Zagreb (05-PA-30-22-11/2023 on 23 November 2023).

*Acinetobacter baumannii* and *Staphylococcus aureus* strains isolated from clinical samples at the University Hospital Centre Zagreb were used to contaminate the dental implants. Oral swabs were inoculated onto a Columbia agar plate enriched with 5% sheep blood (BD Columbia agar with 5% sheep blood; Becton Dickinson GmbH, Heidelberg, Germany). Colonies with distinct morphology were identified as *A. baumannii* and *S. aureus* using matrix-assisted laser desorption/ionization time of flight mass apectrometry—MALDI TOF MS. Susceptibility testing was performed according to the standards of the European Commission for Antimicrobial Susceptibility Testing (EUCAST) [20].

Bacteria were grown separately on Columbia agar enriched with 5% sheep blood under aerobic conditions at a temperature of 35 °C for 48 h. Separate bacterial suspensions were prepared by inoculating one colony of each bacterium separately in 10 mL of brain-hearth infusion (BHI) broth and incubated in aerobic conditions at 35 °C for 24 h. The resulting suspensions were then mixed into a joint suspension containing same volume of *A. baumannii* and *S. aureus.*

**Figure 1:**
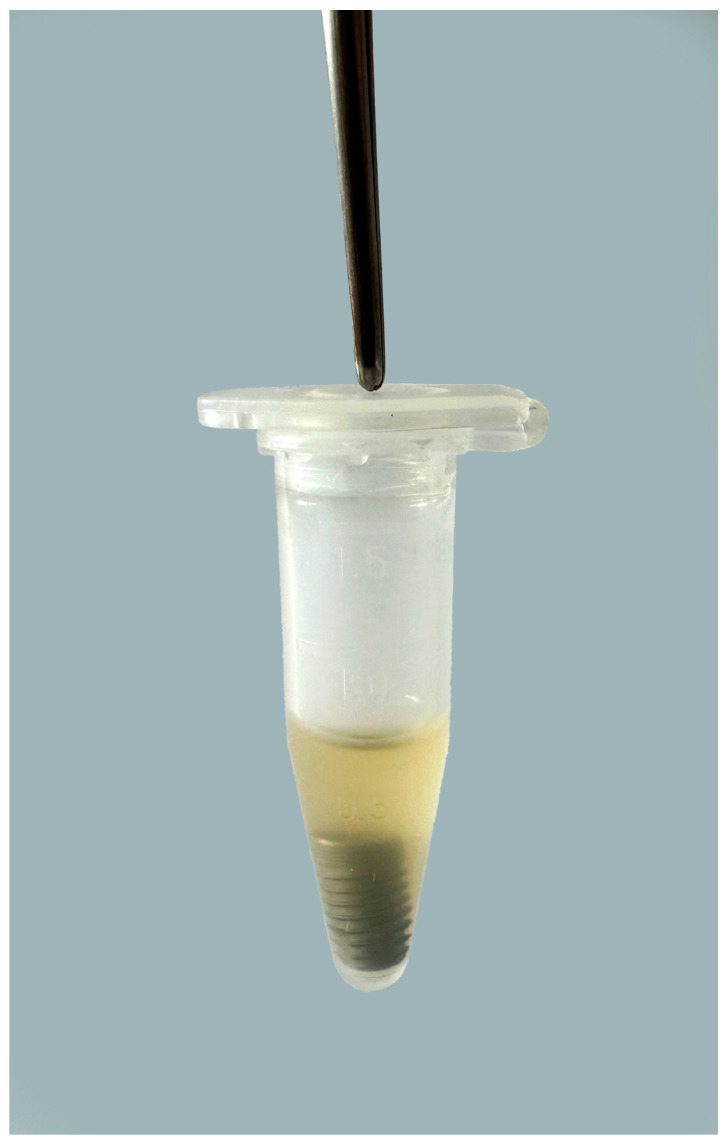
Dental implant placed in test tube containing bacterial suspension of *A. baumannii* and *S. aureus*.

A total of 75 Eppendorf tubes with a volume of 1.5 mL were prepared, to which 500 µL of common bacterial suspension was added. The dental implants were removed from the original sterile packaging with a sterile instrument and placed in test tubes containing bacterial suspension of *A. baumannii* and *S. aureus* (Figure 1).

The test tubes with the suspension and implants were incubated in aerobic conditions at a temperature of 37 °C for 7 days. Every 48 h, 250 µL of fresh, new bacterial suspension was added to improve bacterial survival and suspension stability.

**Figure 2:**
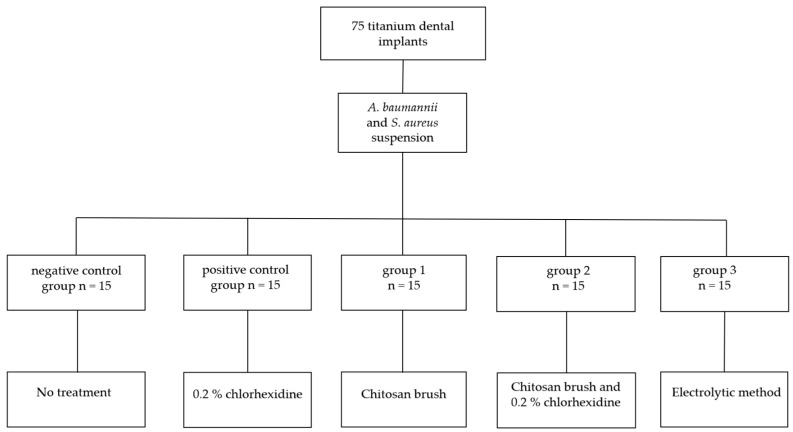
Flowchart showing sample distribution.

The test tubes with the implants were randomly divided into five groups (*n* = 15), a negative control group, a positive control group, group 1, group 2 and group 3, depending on the planned procedure and method of bacterial biofilm removal (Figure 2).

The implants were removed from the bacterial suspension using sterile forceps, washed with sterile saline and gently dried with sterile gauze to remove excess bacterial suspension. The implants were placed on a sterile holder to prevent rotation and movement during sample collection and decontamination. The implants from the negative control group (*n* = 15) were not subjected to decontamination treatment. The implants from the positive control group (*n* = 15) were treated with a 0.2% chlorhexidine solution (Curasept ADS 220, Curaden AG, Kriens, Switzerland). The implant surfaces were continually irrigated for 1 min with a 0.2% chlorhexidine solution using a 20 mL syringe with a 22-gauge needle. The needle tip was kept 3–5 mm from the surface, angled at 60–90° with the implant’s long axis. The surface was then thoroughly washed with sterile saline for 1 min to eliminate any remaining chlorhexidine.

**Figure 3:**
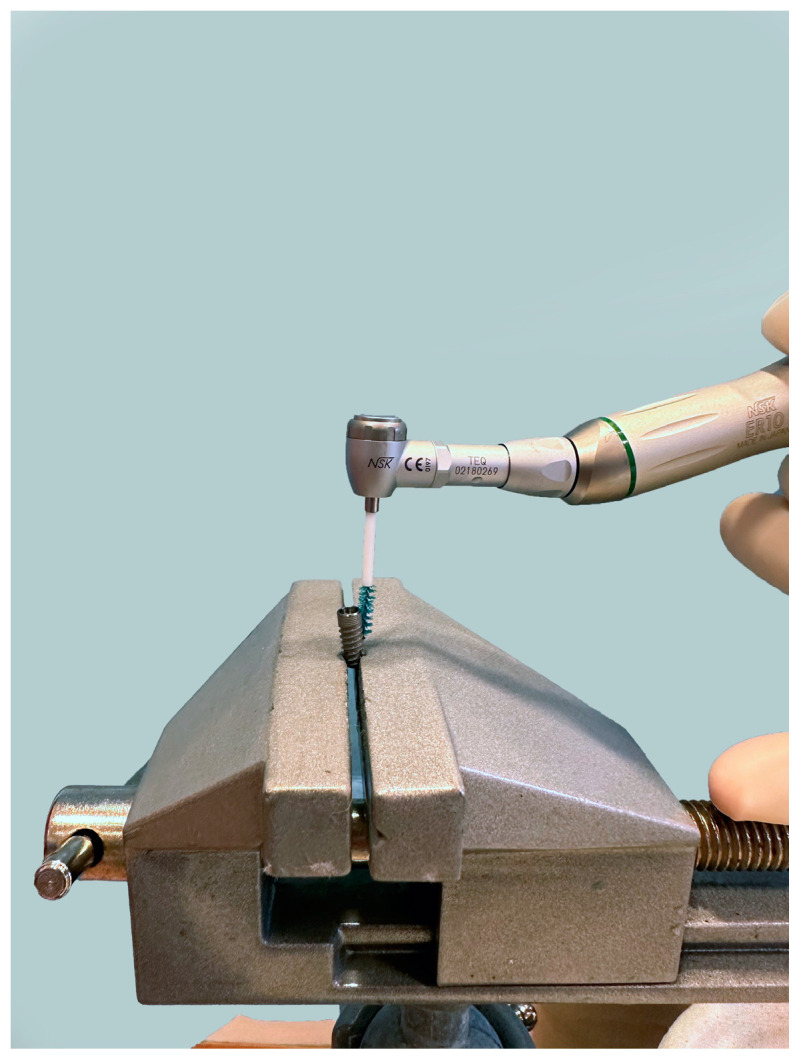
Dental implant treated with a chitosan brush placed on an oscillating contra-angle handpiece.

The implants from group 1 (*n* = 15) were debrided with a chitosan brush (Labrida BioClean™, Labrida AS, Oslo, Norway). After soaking in sterile saline for a duration of two minutes, the brush was mounted on an oscillating contra-angle handpiece (ER10M, TEQ-Y, NSK Inc., Kanuma Tochigi, Japan). The rotation speed was set to 1000 rpm. The brush was used parallel to the long axis of the implant in a gentle manner without using pressure or force (Figure 3). Throughout the entire procedure, contact with the implant was maintained. The implants were treated for two minutes and irrigated with sterile saline after mechanical debridement. The same cleaning procedure was performed in group 2, with additional irrigation with a 0.2% chlorhexidine solution, as in the positive control group.

**Figure 4:**
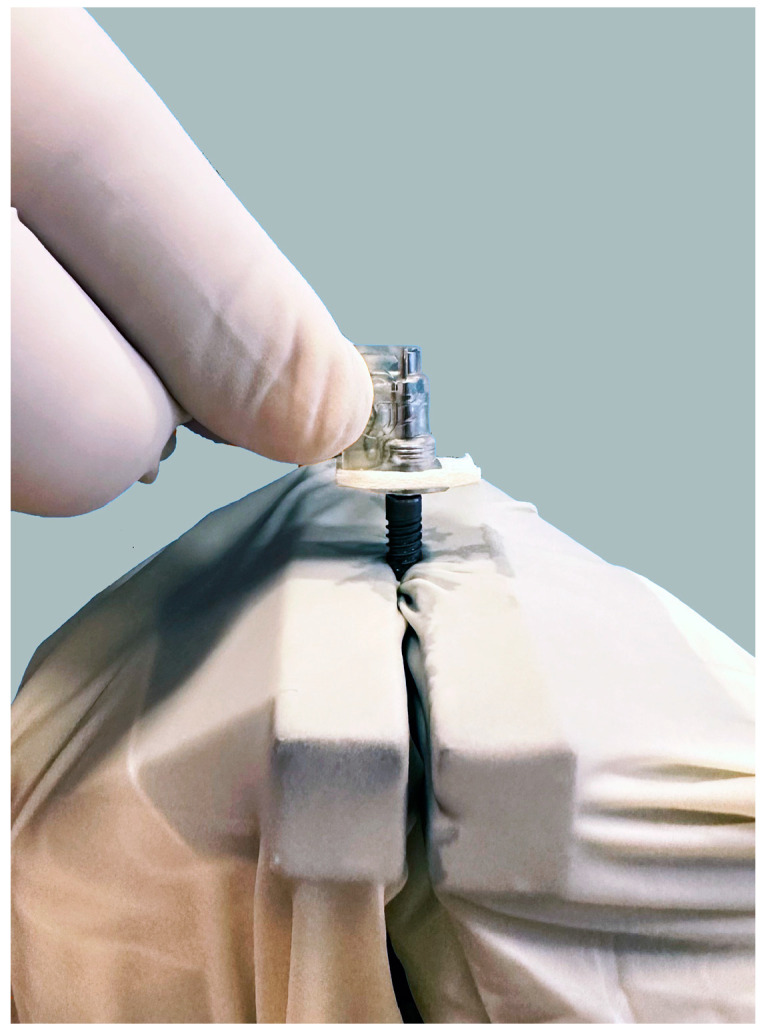
Dental implant treated with a device based on the electrolytic cleaning procedure.

The implants from group 3 (*n* = 15) were treated with a device based on the electrolytic cleaning procedure (GalvoSurge, GalvoSurge Dental AG, Widnau, Switzerland). All preparations before using the device were carried out according to the manufacturer’s instructions. The spray head with the integrated implant connector and the sponge were placed so that the connector was inserted into the interior of the implant and held in place during the two-minute cleaning process (Figure 4). Cleaning solution from the bottle to the spray head was pumped and sprayed evenly around the implant. 

The disinfection of the implant holder was carried out with a 70% ethanol solution and sterile saline after every single implant treatment in order to prevent contamination. During the entire process, sterile instruments and sterile gloves were used to avoid contamination of the implant with bacteria that were not part of the biofilm.

Samples were collected using sterile paper swabs, with five horizontal strokes between the second and fifth threads of the implant (viewed from coronal to apical). Each paper swab was immersed in a separate Eppendorf tube containing 200 µL of phosphate-buffered saline (PBS). Each tube was then vortexed (Corning LSE vortex mixer, Corning, NY, USA) for 40 s at a speed of 2850 rpm.

Serial dilutions were carried out in microtiter plates. A total of 20 μL of vortexed PBS was added to 180 μL of Mueller–Hinton (MH) broth (Mueller–Hinton II Broth, Becton Dickinson GmbH, Heidelberg, Germany), and 20 μL was transferred to the next well. Serial dilution up to 1 × 10^−8^ was performed. From each well, 20 μL of MH broth was transferred to a previously labelled section of a blood agar plate. Undiluted PBS was transferred to the labelled section in the middle of the blood agar.

The blood agar plates were incubated under aerobic conditions at a temperature of 37 °C for 48 h, after which visible viable bacterial colonies were counted. The resulting bacterial colonies were counted by visual inspection by an experienced researcher. The researcher who counted the colonies was not familiar with the method of decontamination on the observed sample. Macroscopically different colonies from 15 randomly selected blood agar plates were confirmed using a MALDI Biotyper device (Bruker Daltonics, Hamburg, Germany), and the obtained results were entered into prepared tables.

### Statistical Analysis

Microsoft Excel data analysis tools (Microsoft Corporation, Redmond, WA, USA, from https://office.microsoft.com/excel, accessed on 12 December 2023) were used for the statistical data analysis. The statistical analyses were performed using analysis of variance (ANOVA) with a statistical significance level set to 0.05 (5%).

## 3. Results

**Figure 5:**
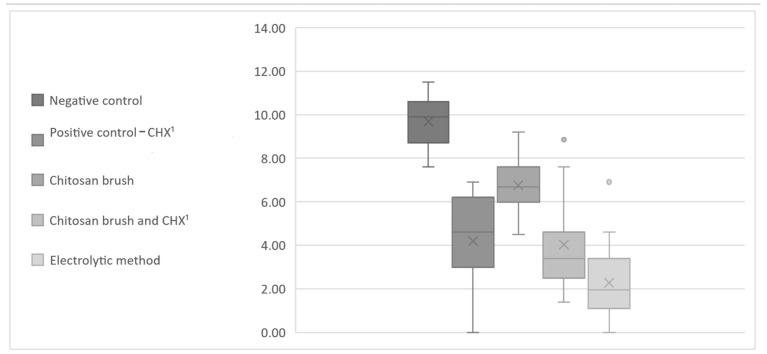
Box-and-whisker chart presenting the logarithm of CFU counts (*A. baumannii*). ^1^ Chlorhexidine.

**Figure 6:**
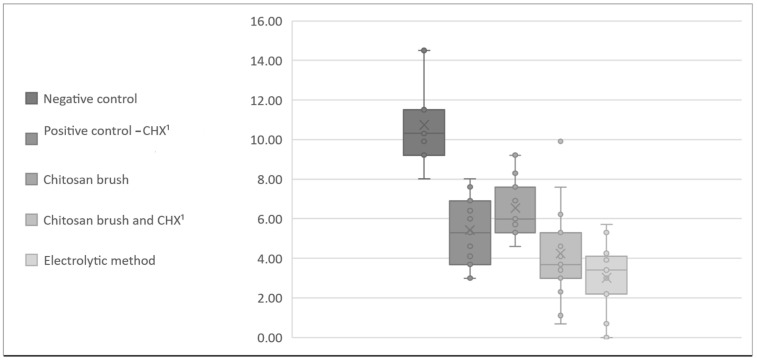
Box-and-whisker chart presenting the logarithm of CFU counts (*S. aureus*). ^1^ Chlorhexidine.

Descriptive statistics of the CFU count for all the methods for *A. baumannii* and *S. aureus* are presented in Table 1 and Table 2. The data from Table 1 and Table 2 were transformed using the logarithm of the CFU counts in order to illustrate the differences between the control and test groups (Figure 5 and Figure 6).

ANOVA was used to determine whether there were any statistically significant differences between the negative control group and all the decontamination methods for the bacteria. There was a statistically significant difference in the number of bacterial colonies on the surface of the untreated implants in the control group and the implants that underwent decontamination methods.

The next step was to determine if there was any significant decontamination effect between all the aforementioned decontamination methods. The ANOVA test was used to analyze the differences between the methods, and the results are shown in Table 3 and Table 4.

An additional ANOVA test was used to test the relationships between each pair of methods in order to determine the dominant decontamination method. For all pairs of decontamination methods, the p-values of the ANOVA test are presented in Table 5 and Table 6. Table 5 and Table 6 show that the electrolytic decontamination method significantly reduced the CFU count when compared to all the other methods for *A. baumannii*, as well as the positive control (0.2% chlorhexidine solution) and the chitosan brush for *S. aureus* (*p*-values were lower than 5%). 

Considering the objectives of this study, there was a statistically significant difference between the methods for the removal of the *A. baumannii* and *S. aureus* biofilms. Given that we witnessed some higher standard deviation values, we transformed the original CFU data with the logarithm function. Using the original CFU data (not logarithm-modified), we might expect the electrolytic cleaning method to be more effective than the other three treatments in a larger study.

## 4. Discussion

In modern dental medicine, there are several different procedures and protocols for the removal of biofilm and the prevention and treatment of periimplantitis [3,5]. If reosseointegration and soft tissue reattachment are to be achieved, it is desirable that the decontamination process does not damage or negatively affect the surface of the implant. In similar studies [21,22,23], it was found that titanium brushes and curettes cause damage and changes to the surface of the titanium discs and implants, so we decided to use chitosan brushes in this study, which are not expected to cause negative changes to the surface of the implant. Except for chitosan brushes, the same was established for the electrolytic cleaning method [23], so the methods compared in our research were not expected to cause damage to the implant surface.

The bacteria *A. baumannnii* and *S. aureus* were used in this study because of their propensity to create biofilm, which gives them the ability to survive on different surfaces and in different conditions [24,25,26]. In addition to being associated with periimplantitis, periodontitis and pulp and root canal infections, these bacteria also cause purulent skin infection, infections of surgical wounds, osteomyelitis, pneumonia and endocarditis [24,26,27,28]. The World Health Organization classified *A. baumanni* and *S. aureus* in the group of so-called ESCAPE pathogens with *Enterococcus faecium*, *Clostridiodes difficile*, *Pseudomonas aeruginosa* and *Enterobacteriaceae* [24,26,29]. The aforementioned bacteria are resistant to numerous antimicrobial drugs; they cause serious hospital infections and represent a significant health and financial problem to public health [24,25,26]. The results obtained from this research could perhaps be useful in the treatment of similar infections by these two bacteria in other medical specialties, such as orthopaedics and traumatology.

In their research, Alagl et al. [30] also contaminated implants with two bacteria from the ESCAPE group, *A. baumannii* and *P. aeruginosa*, to obtain a biofilm. In contrast to this study, Alagl et al. examined the effectiveness of an erbium laser, photodynamic therapy, a diode laser and a 0.12% chlorhexidine solution. Although different concentrations of chlorhexidine were used, i.e., in their research, 0.12%, and in ours, 0.20%, the chlorhexidine reduced the number of bacteria on the implant surface. Afrasiabi et al. showed that the combined use of hydrogen peroxide with a photosensitizer and photodynamic therapy has a more efficient antibacterial effect on biofilms of *Staphylococcus aureus*, *Escherichia coli* and *Candida albicans* compared to each treatment alone [31]. Similarly, Sousa et al. [22] achieved better results with chemical treatment with sodium hypochlorite and chlorhexidine after mechanical cleaning with a titanium brush. The results of the aforementioned studies show that using a chemical agent in addition to mechanical biofilm removal can improve the decontamination of the implant surface [22,30,31]. This is in accordance with the results of our study, where after treatment with a chitosan brush and chlorhexidine, fewer bacteria were found than if a chitosan brush was used alone. Although a high efficiency of chlorhexidine was observed in in vitro studies, Monje et al. state that no clinical benefit was demonstrated [3]. They further state that the existing evidence does not support the use of chlorhexidine as an adjunctive agent to promote implant decontamination or reosseointegration due to its osteoblast cytotoxicity [3]. Regarding that, a sulfonic/sulfuric acid solution may be used as an alternative to chlorhexidin. In Citterio et al.’s [32] study, a sulfonic/sulfuric acid solution in a gel demonstrated great potential against polymicrobial biofilms (*Staphylococcus aureus*, *Staphylococcus epidermidis*, *Streptococcus anginosus*, *Streptococcus salivarius*, *Streptococcus mitis*, *Fusobacterium nucleatum* and *Capnocytophaga ochracea*) on titanium surfaces. They also observed that the sulfonic/sulfuric acid solution did not alter cell morphology, suggesting that it may have no or limited cytotoxic activity when compared to chlorhexidine [32].

In addition to the chemical agent itself and its effect on osteoblasts and other cells, the way it is applied to the surface of the implant is also very important. Ichioka et al. [33] used gauze soaked in saline in their research as one of the procedures for removing biofilm from the surface of titanium discs. They found that this type of cleaning proved to be inferior to other methods in restoring cytocompatibility. As one of the possible reasons for this, they mentioned the remains of gauze fibres and deposits of foreign material on the treated surface of the disc [33]. This could also be a problem in the clinical application of any liquid chemical agent and the achievement of reosseointegration and soft tissue reattachment. One solution could be to avoid the application of chemical agents in liquid form using gauze or cotton wool on the rough surfaces of the implant and to choose a more suitable method of decontamination with liquid chemical agents.

In comparison with the negative control group, i.e., implants in which no biofilm removal procedure was performed, in the treated implants under the conditions of the presented research, all four procedures significantly reduced bacterial contamination. This indicates that chlorhexidine, a chitosan brush, a chitosan brush and chlorhexidine and the electrolytic method, regardless of the differences between them, can remove bacteria from the grooves, threads and rough surface of titanium dental implants. This is in accordance with similar in vitro studies by other authors who compared different decontamination procedures [13,21,22,30,34]. Some of these procedures are simpler and do not require expensive devices, while others are more complex and more expensive. Regardless of the differences in the decontamination procedures and the difference in the effectiveness between them, performing any of them is better than leaving the biofilm untreated on the implant surface.

In the research of Zipprich et al. [13], the electrolytic method proved to be the most effective of the tested methods. Although different methods were tested and the biofilm was formed differently, even in the conditions of this research, the electrolytic method proved to be the most effective. The reason for this could be that an activated electrolytic cleaning solution has easier access to the threads and the rough surface of an implant, unlike brush fibers and syringe-irrigated chlorhexidine. The limitations of the electrolytic method are that it can only be performed on electrically conductive dental implants and that the prosthetic components and implant abutments must be removed preoperatively.

The advantages of this study include the use of original implants, while titanium discs were used in similar studies [22,33,34]. The implant’s complex geometry more accurately mimics the clinical scenario of implant decontamination than titanium discs. Two types of bacteria were used, while in similar studies, one bacterial monoculture was used [23,33,34]. The limitations of this study are similar to those of other in vitro studies, including easy accessibility and the ability to clean the contaminated surfaces from all angles. As a limitation, we mention the use of the biofilm of two bacterial strains as opposed to a biofilm formed by the colonization of wide spectra of bacteria in clinical situations. Using one brand of implant is also a limitation, as there are many implant systems with different surfaces available on the market today.

## 5. Conclusions

According to the presented results, all four examined methods reduced the number of bacteria on the implant surface in in vitro conditions. The electrolytic method proved to be the most effective, and the chitosan brush was the least effective in the removal of *A. baumannii* and *S. aureus* biofilms. Considering the discussed limitations of this study, the electrolytic method represents a viable solution from a microbiological point of view. Further research into more realistic in vitro and in vivo conditions is needed to evaluate the results of the decontamination methods reported in this study.

## Figures and Tables

**Table 1:** *A. baumannii* colony-forming units according to groups.

CFU *A. baumannii*	Negative Control	Positive Control (CHX ^2^)	Chitosan Brush	Chitosan Brush and CHX ^2^	Electrolytic Method
avg CFU	30,867	276	1906	645	83
min CFU	2000	0	90	4	0
max CFU	100,000	1000	10,000	7000	1000
sd ^1^	33,233	361	2959	1829	255
median CFU	20,000	100	800	30	7

^1^ Standard deviation; ^2^ chlorhexidine.

**Table 2:** *S. aureus* colony-forming units according to groups.

CFU *S. aureus*	Negative Control	Positive Control (CHX ^2^)	Chitosan Brush	Chitosan Brush and CHX ^2^	Electrolytic Method
avg CFU	303,533	705	2060	1542	58
min CFU	3000	20	100	2	0
max CFU	2,000,000	3000	10,000	20,000	300
sd ^1^	689,789	931	3379	5131	84
median CFU	30,000	200	400	40	30

^1^ Standard deviation; ^2^ chlorhexidine.

**Table 3:** ANOVA single-factor test, decontamination methods—*A. baumannii*.

SUMMARY						
*Groups*	*Count*	*Sum*	*Average*	*Variance*		
Positive control (CHX ^1^)	15	63.03	4.20	5.04		
Chitosan brush	15	101.46	6.76	1.56		
Chitosan brush and CHX ^1^	15	60.53	4.04	4.07		
Electrolytic method	15	34.04	2.27	3.63		
ANOVA						
*Source of Variation*	*SS * ^2^	*df * ^3^	*MS * ^4^	*F*	*p*-value ^5^	*F crit*
Between groups	154.13	3	51.378198	14.361941	4.69 × 10^−7^	2.769431
Within groups	200.33	56	3.577385			
Total	354.47	59				

^1^ Chlorhexidine, ^2^ the sum of squares due to the source, ^3^ the degrees of freedom in the source, ^4^ the mean sum of squares due to the source, ^5^ *p*-*value* < 0.05.

**Table 4:** ANOVA single-factor test, decontamination methods—*S. aureus*.

SUMMARY						
*Groups*	*Count*	*Sum*	*Average*	*Variance*		
Positive control (CHX ^1^)	15	81.38	5.43	3.05		
Chitosan brush	15	98.24	6.55	2.18		
Chitosan brush and CHX ^1^	15	63.59	4.24	5.60		
Electrolytic methode	15	45.16	3.01	3.09		
ANOVA						
*Source of Variation*	*SS * ^2^	*df * ^2^	*MS * ^4^	*F*	*p*-value ^5^	*F crit*
Between groups	104.51	3	34.83733	10.00527	2.23 × 10^−5^	2.76943
Within groups	194.99	56	3.48190			
Total	299.50	59				

^1^ Chlorhexidine, ^2^ the sum of squares due to the source, ^3^ the degrees of freedom in the source, ^4^ the mean sum of squares due to the source, ^5^ *p*-value < 0.05.

**Table 5:** *A. baumannii* ANOVA test *p*-values for pair comparison of expected CFUs.

ANOVA (*p*-Values)	Positive Control (CHX ^1^)	Chitosan Brush	Chitosan Brush and CHX ^1^	Electrolytic Method
Positive control (CHX ^1^)		0.0006	0.8324	0.0169
Chitosan brush		0.0001	2.54 × 10^−8^	0.0322
Chitosan brush and CHX ^1^			0.0201	0.2568
Electrolytic method				

^1^ Chlorhexidine.

**Table 6:** *S. aureus* ANOVA test *p*-values for pair comparison of expected CFUs.

ANOVA (*p*-Values)	Positive Control (CHX ^1^)	Chitosan Brush	Chitosan Brush and CHX ^1^	Electrolytic Method
Positive control (CHX ^1^)		0.0674	0.1297	0.0008
Chitosan brush			0.0033	1.98 × 10^−6^
Chitosan brush and CHX ^1^				0.1177
Electrolytic method				

^1^ Chlorhexidine.

## Data Availability

The data presented in this study are available on request from the corresponding author.
